# Influence of the Cellulose Purification Method on the Properties of PVA Composites with Almond Shell Fibres

**DOI:** 10.3390/molecules30020372

**Published:** 2025-01-17

**Authors:** Irene Gil-Guillén, Chelo González-Martínez, Amparo Chiralt

**Affiliations:** Institute of Food Engineering-FoodUPV, Universitat Politècnica de València, 46022 Valencia, Spain; cgonza@tal.upv.es

**Keywords:** polyvinyl alcohol, thermoprocessed composites, almond shell cellulose, radical scavenging, oxygen barrier, water vapour permeability, tensile properties, thermal properties

## Abstract

Almond shells (ASs) are a potential source of cellulose that could be obtained through sustainable methods for their valorisation. Biocomposites (BCs) from polyvinyl alcohol (PVA) and cellulose are interesting materials for developing sustainable packaging materials. BC based on PVA and AS cellulose were obtained by melt blending and compression moulding, by using subcritical water extraction at 160 or 180 °C, and subsequent bleaching with sodium chlorite (C) or hydrogen peroxide (P) to purify cellulose. The influence of the purification method on the properties of BC was analysed. Fibres treated with C were better dispersed in composites than those bleached with P. Residual phenolic compounds in the fibres provide the composite with ABTS∙+ scavenging capacity in line with the residual lignin content of the fibres. Both the presence of phenols and dispersed fibres reduced the film transparency, mainly in the UV range. Fibres enhanced the oxygen barrier capacity of composites, and those treated with HP also improved the water vapour barrier capacity. Fibres treated with C better promoted the increase in the elastic modulus of the composites, due to their highest crystallinity and dispersibility, while favoured the PVA crystallisation. Therefore, the obtained AS cellulose fibres could be used to obtain thermoprocessed PVA biocomposites for food packaging applications.

## 1. Introduction

In the last ten years, the almond (*Prunus dulcis* (Mill.) DA Webb) has become the most produced nut in the world. The worldwide almond production was 1.5 Mt in 2021 [1], and almond cultivation generates between 70 and 150 million tons of almond shells annually [2]. Almond production results in the generation of solid by-products, such as shells, hulls, and skins, as well as liquid by-products, such as the blanching water from the industrial processing of the nut. These by-products are mostly utilised for the manufacture of biofuel and animal feed [3]. However, their composition makes them an interesting source of high-value bioactive phytochemicals, such as flavonoids, phenolic acids, and condensed and hydrolysable tannins [4] and polymers such as cellulose or lignin [5].

Nowadays, an increasing amount of research is focused on employing biodegradable materials to replace fossil fuel-based products, reducing plastic pollution, and encouraging environmentally friendly, sustainable developments [6,7]. Almond shell (AS), whose main constituents are cellulose, hemicellulose, and lignin, has been studied as to its potential for use in different processes, including the development of affordable bioadsorbents for contaminated solutions [8,9], as a reinforcing agent in a variety of polymer matrices, including polypropylene [10,11], polylactic acid [12,13], or starch-based biocomposites. This agricultural waste has been identified as a renewable natural resource for the extraction of biodegradable polymers, such as cellulose [14,15,16], which constitute 20–38% of the product, depending on the origin, culture practices and variety [17].

Different methods to extract/purify cellulose from AS have been used by several authors [18,19]. These include various chemical treatments, such as solvent extraction, alkaline or acid hydrolysis and bleaching treatments, mainly with sodium chlorite, or TEMPO-oxidising [20,21,22], which produce effluents with high pollutant load. However, for both economic and environmental reasons, it is essential to recover chemicals from agri-food waste by employing more sustainable and green methods of extraction, reducing chemicals [23]. In this sense, Valdes et al. applied microwave-assisted extraction of cellulose [24] from AS waste to optimise the use of chemicals. Gil-Guillén et al. [25] developed a more environmentally friendly method consisting of subcritical water extraction (SWE) at 160 or 180 °C to partially extract non-cellulosic compounds, such as hemicellulose of a part of the phenolic fraction, without using chemicals, followed by a bleaching step with hydrogen peroxide as a greener oxidising agent than sodium chlorite, which is recalcitrant to degradation. In this study, cellulose fibres from AS with different purity and morphology were obtained, depending on the SWE extraction temperature and the type of bleaching agent. These fibres could be used to obtain biocomposites, but the effect of their differences on the material properties should be analysed.

Biocomposites are an interesting alternative for developing innovative and biodegradable materials for the growing sector of sustainable packaging [26], which include nanopapers, biocomposites, and bionanocomposites [27,28]. Biocomposites based on biodegradable polymers and natural fibres are considered an environmentally friendly alternative to petroleum-based, non-biodegradable polymeric materials [29,30], since these are biodegradable and low cost, with low density, and good thermal and mechanical properties. Their use as food packaging materials also requires an effective barrier to oxygen and water vapour, as well as mechanical performance, heat sealability, print quality, and UV resistance. Antioxidant and antibacterial properties would also be appreciated to ensure food safety standards [31].

Of the biodegradable polymers, polyvinyl alcohol (PVA) is a commonly utilized petroleum-based thermoplastic polymer [32]. It is a semi-crystalline hydrophilic polymer that is chemically and thermally stable, with excellent film-forming capacity, giving rise to transparent films with high mechanical strength. It is also non-toxic and biocompatible, with reactive -OH groups that make the formation of different blends and composites easier. Because of these characteristics, it is widely used in different fields, including electrical insulation, packaging, or biomedicine [33]. Due to its physicochemical properties, such as emulsifying or adhesive capacity, or water solubility, it has been widely used in composite formulations [34]. PVA/cellulose-based nanocomposites have shown excellent structural stability, enabling their use in a variety of industries, including the biomedical, packaging, and textile sectors, or in heavy metal removal [35,36,37]. Low-molecular-weight organic substances, such as sorbitol, glycerol, and polyethylene glycol, are frequently employed as plasticisers, but glycerol has advantages in producing biocomposites [38]. Many authors analysed the properties of cellulose–PVA composites obtained by casting the water dispersions of the polymer and nanocellulose fractions [30,39,40,41]. Nevertheless, the thermoplastic processing (melt blending, extrusion, …) of the polymer and composites has the advantage of being a continuous manufacturing process more applicable on an industrial scale, without the requirement of further drying steps. On the other hand, no previous studies were found using AS cellulose in the formulation of PVA composites.

Selection of the PVA polymer grade is important to ensure its thermal stability during thermoprocessing and the performance of the obtained films. There are several PVA grades on the market, with different molecular weights and hydrolysis degrees, which provide the materials with different physical properties. PVA with high molecular weight improves tensile strength and elongation at the break of the films, while a high hydrolysis degree of the PVA leads to more rigid films [42]. Likewise, the acetylation degree improves the thermal stability of the polymer, opening the processing window for thermal processing since the difference between melting and degradation temperature increases [43]. In the cellulose–PVA composites, the good dispersion of the fibres in the molten polymer constitutes a critical point that will affect the film properties [44].

The aim of this study was to obtain PVA biocomposites with almond shell cellulose, analysing the influence of the cellulose purification method on the properties of the films. Four types of cellulose purification treatments were considered, combining a first extraction step with subcritical water at 160 or 180 °C, and a second bleaching step consisting of a more environmentally friendly treatment with hydrogen peroxide or the usual treatment with sodium chlorite. The biocomposites were obtained by melt blending and compression moulding and their functional properties for food packaging uses, such as optical, tensile, water vapour and oxygen barrier properties, were analysed. Furthermore, to better explain the properties of the composites, their microstructure and the polymer thermal behaviour and stability were characterised.

## 2. Results and Discussion

### 2.1. Structural and Optical Properties of the Films

Table 1 shows the composition and whiteness index of the used fibres, compared to the values of raw AS, as reported in a previous study [25], to better understand their effect on the properties of composites. As previously reported, SWE fibres treated at 160 °C had higher hemicellulose content than those treated at 180 °C while treatment with sodium chlorite gave rise to a higher delignification degree, also reflected on the greater values of the fibre whiteness index. The fibre with the highest cellulose purity and whiteness was treated at 180 °C and sodium chlorite. The treatment also affected the fibre morphology [25]. The samples treated with sodium chlorite were more regular and elongated in shape, whereas those bleached with hydrogen peroxide showed a less uniform aspect with a more unstructured surface. In every case, AS fibres exhibited small pores clearly observed along their surface [25].

**Figure 1:**
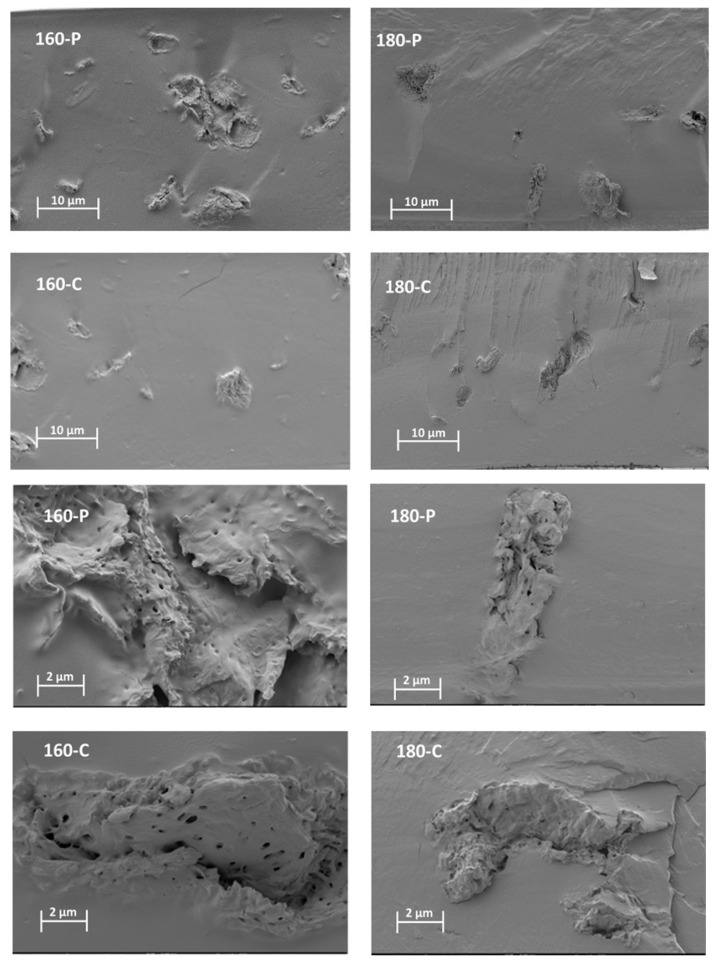
FESEM micrographs at different magnifications (×400, top and ×2000, bottom) of the PVA films with different AS cellulose particles obtained by SWE at 160 or 180 °C and bleached with hydrogen peroxide (P) or sodium chlorite (C).

**Figure 2:**
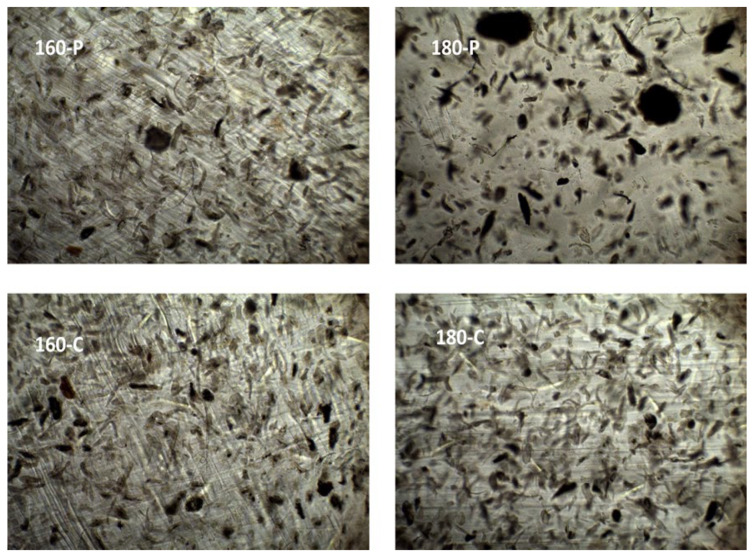
Light microscopy images (×10) of the PVA composites with different AS cellulose particles obtained by SWE at 160 or 180 °C and bleached with hydrogen peroxide (P) or sodium chlorite (C).

The different morphological features previously described for the fibres could also be observed when these were embedded within the PVA matrix. Figure 1 shows the FESEM images of the cross-section of the composites, at different magnification levels, where the dispersed cellulose particles can be observed. The micrographs clearly showed differences in the particle dispersibility within the composite matrix. The fibres treated with oxygen peroxide were more aggregated than those treated with sodium chlorite, which appears more homogeneously dispersed as individual particles. This can be better appreciated in Figure 2, where the light microscopy images of the different composites are shown, offering a wider field of observation of the films. In every case, very good interfacial adhesion between fibres and PVA matrix could be deduced, since no gaps were observed in the surrounding fibre area while fibre appeared cross-fractured or coated with a densely adhered polymer layer that partially covered the porous surface of the fibres. In some cases, the imprint of the fibre pores can be seen in the voids in the matrix left by the fibre detachment. This suggests the penetration of the polymer into the fibre pores, which probably contributed to the good adhesion between fibres and polymer. Therefore, the main difference in the microstructure of the composites comes from the higher aggregation tendency of the hydrogen peroxide-treated fibres, which contained more lignin and less cellulose (Table 1).

The different treatments applied to the fibres implied the modification of their overall composition, size, and shape, and surface properties, due to the different degrees of elimination of non-cellulosic compounds such as lignin, hemicellulose and other minor components of the fibre structure. Morphological properties of the fibres can also affect the properties of composites. The combination of all different factors could result in a fibre effect that is difficult to predict based on a single factor, such as cellulose content. These differences will affect their aggregation capacity and the dispersion in the polymer matrix as well as the interfacial adhesion within the polymer matrix of composites [44].

The presence of dispersed particles into the PVA matrix and the potential diffusion of low-molecular-weight compounds from the fibres during the melt blending process affected the light interactions with the matrix and so the film colour and transparency. Colour coordinates of the films are shown in Table 2 where the different effects of the fibres can be observed.

**Figure 3:**
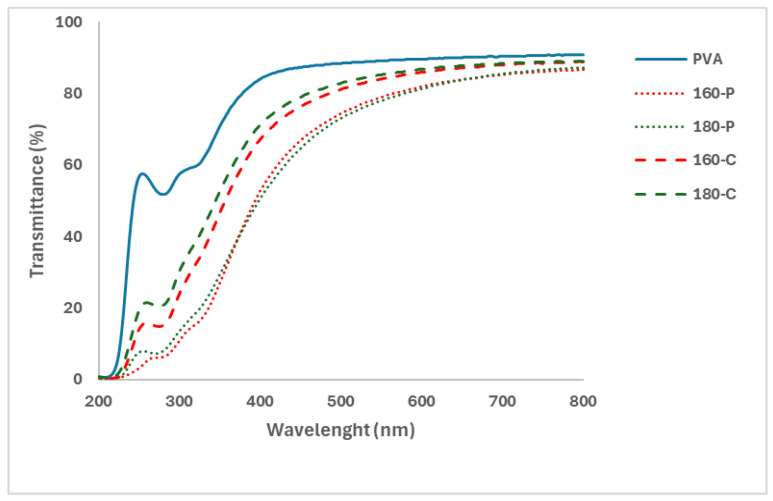
UV-Visible transmittance spectra of the PVA films without and with different AS cellulose particles obtained by SWE at 160 or 180 °C and bleached with hydrogen peroxide (P) or sodium chlorite (C).

Likewise, Figure 3 shows the UV-Visible spectra of the different films where the changes in light absorption produced by the different fillers are shown. Fillers produced a decrease in the light transmission of the PVA films mainly in the UV region. This decrease in light transmission must be attributed to the presence of phenolic compounds present in the fibres (residual lignin), which mainly absorb in this wavelength range, and to the light scattering effect produced by dispersed particles with different refractive index to that of the PVA matrix. The light scattering effect, described by Maxwell’s equations depends on the particle size and shape and the wavelength, the relative size of a scattering particle being defined by its size parameter, which is the ratio of its characteristic dimension to the wavelength. For particles with critical dimensions much larger than the wavelength of light, which is considered a collection of rays, each ray hitting the particle may undergo (partial) reflection and/or refraction, thus affecting the reflection and transmission spectra of the material [45]. In this sense, fillers treated with hydrogen peroxide that appear more aggregated in the PVA matrix would produce more light scattering effects, reducing light transmission. In these samples, a higher content of lignin and phenolic compounds would also lead to greater light absorption in the UV region, as observed in Figure 2. There were no noticeable differences associated with the extraction temperature applied at the SWE stage, which mainly affected the final hemicellulose content of the filler. Therefore, films with fibres treated with hydrogen peroxide could better protect the packaged product from light-induced oxidation reactions due to their higher opacity in the UV region.

The colour coordinates of the composites also reflected the light absorption and scattering effects of the different films. The lightness (L* values) of composites decreased with respect to the net PVA films, whereas the colour became more saturated (higher C_ab_* values) and yellowish (higher h_ab_* values). The increase in the colour saturation and the lightness decrease was more marked for composites containing fibres bleached with hydrogen peroxide. This can be attributed to their higher lignin content (Table 1) and the corresponding light absorption in the red wavelength region (400–500 nm) of the spectra (Figure 3).

**Figure 4:**
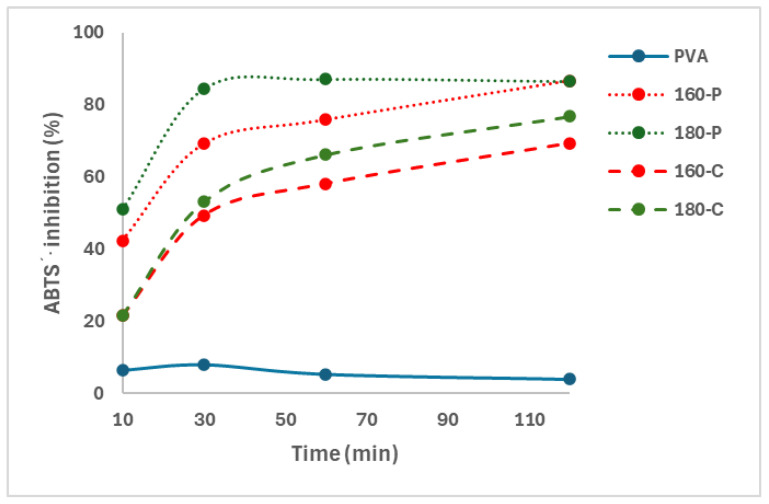
Inhibition of the ABTS∙+ radical by the PVA films without and with different AS cellulose particles obtained by SWE at 160 or 180 °C and bleached with hydrogen peroxide (P) or sodium chlorite (C).

The presence of phenolic compounds in the composite films was demonstrated through their antioxidant capacity measured through the ABTS∙+ radical scavenging capacity. Oxidation reactions occur through complex mechanisms involving the formation of free radicals, so the antioxidant capacity of different compounds is usually analysed through their ability to inhibit different free radicals, such as DPPH or ABTS∙+ [46]. Figure 4 shows the percentage inhibition of the different films as a function of the film contact time with the radical solution. The progressive release of active compounds with the ability to scavenge the radical ABTS∙+ in the solution could be deduced from the increase in percentage inhibition as the contact time rose. Therefore, these compounds could migrate from the fibres into the PVA matrix, mainly during the melt blending process at high temperatures, which would also affect the properties of composites through interactions with the polymer chains by forming hydrogen bonds and affecting the chain packing in the matrix. Phenols bound to the fibres may also exert antioxidant activity by quenching the ABTS∙+ radicals of the solvent phase, diffusing to the fibre surface and reacting at the interface [47].

### 2.2. Barrier Properties of the Films

Given the hydrophilic nature of the PVA, the oxygen and water vapour barrier capacity of the films will be greatly affected by the plasticising effect of the water molecules, depending on the equilibrium moisture content of the films conditioned at a determined relative humidity (RH). The equilibrium moisture content of the PVA films at 53% RH (Table 3) slightly decreased when the cellulose particles were incorporated, which can be attributed to the lower water binding capacity of the fibres constituted by closely packed cellulose chains through interchain hydrogen bonds, with lower water binding capacity. Diffusion of phenolic compounds from the fibres into the PVA matrix could also modify its water-binding capacity through the formation of hydrogen bonds with the hydroxyl groups of the polymer chains, blocking these active points for water binding. The reduction in the equilibrium moisture content was slightly more marked for fibres 160-P, which have the highest content of lignin whose phenolic compounds could slightly modify the hydrophilic nature of the PVA matrix. This property can also be affected by the molecular characteristics of the polymer (molecular weight and hydrolysis degree) as well as by the plasticiser used (kind and content). Andrade et al. [48] reported an equilibrium moisture content of 6%, for thermoprocessed PVA films with 9% wt. of glycerol, conditioned at 53% RH, using a polymer with similar hydrolysis degree but lower mean molecular weight. This water content also slightly decreased when phenolic acids were incorporated at 1–2% into the matrix.

In contrast, the contact angle values of the films (Table 3) did not reflect significant changes in the hydrophobicity of the film surface by fibre incorporation, since these were not significantly affected by the incorporation of fibres. Nevertheless, these values exhibited high variability due to different factors, such as the high solubility of the water drop onto the film surface that makes their analysis difficult, and the heterogeneous surface roughness of the thermoprocessed films. Different values of contact angles have been reported for PVA films obtained by casting, in the range of the obtained values. Jayasekara et al. [49] reported 44°, whereas Nuruddin et al. [50] reported 35°, increasing to 45° when films contained 10–30% of cellulose nanocrystals. The molecular characteristics (molecular weight and hydrolysis degree) of the polymer as well as the film processing method can affect the chain orientation at the surface level, thus affecting the surface hydrophobicity. The casting method allows for better chain interactions in the film, due to the greater chain unfolding in solution and oriented aggregation in the matrix. In the film thermoprocessing, these mechanisms are more hindered by the high viscosity of the melt [48].

Water vapour and oxygen permeability of the films are also shown in Table 3, where the effect of the fibres on the barrier capacity of composites can be observed. The OP values obtained for net PVA films were in the range obtained by other authors [48,50,51]. The OP values were significantly reduced (by about 75%) by the cellulose particles obtained at 160 °C that contained 20% of hemicellulose, whereas the reduction was lower (by about 40–65%) for fibres treated at 180 °C. Other authors [50] also observed a reduction in OP values of PVA films by about 50%, when CNC were incorporated into the polymer matrix. This reduction can be attributed to the promotion of the tortuosity factor for oxygen transport by the cellulose particles that are less permeable than the plasticized PVA. In contrast, a different effect was observed for WVP, where cellulose fibres bleached with hydrogen peroxide promoted the water vapour barrier capacity of the films (by 17–26%), whereas in those bleached with sodium chlorite, this capacity decreased (by 22% in sample 160-C) or did not change (no significant differences for sample 180-C). Diffusion and solubility of water molecules through the cellulose particles could be affected by their composition and morphology, which would modify their effective tortuosity factor for the transport of the water molecules through the films. Likewise, diffusion of phenolic compounds from the lignin-rich fibres within the PVA matrix and the formation of hydrogen bonds between the phenol-hydroxyl groups could also affect the transport properties of the polymer matrix. Thus, fibres treated with hydrogen peroxide with higher lignin content seem to better limit the transport of water molecules through the PVA matrix, whereas the higher content of hemicellulose in 160-C fibres could promote the transport of water molecules through the filler. The different dispersion degree of the particles in the polymer matrix and their interfacial adhesion forces will also affect the transport properties of the composite. Other authors [50] observed an increase in the WVP of PVA films containing CNC that was attributed to the high hydrophilic nature of cellulose. In this case, the absence of residual lignin, which could modulate the properties of the polymer matrix, resulted in no improvement in the water barrier capacity of the composites.

Therefore, different factors affected the mass transport properties of the PVA–cellulose composites: (1) the fibre components and their potential diffusion within the polymer matrix affecting the interchain forces, (2) the particle size and dispersion degree that affects the tortuosity factor in the matrix and, (3) the crystallinity degree of the fibres, depending on the cellulose purity, which also affect their permeation capacity. In this sense, Gil et al. [25] found a higher crystallinity degree for fibres bleached with sodium chlorite (64–69%) than for those treated with hydrogen peroxide (50–62%). 

### 2.3. Tensile Properties of the Films

Table 4 shows the tensile properties of the different PVA films with and without cellulose fillers. Tensile proprieties of PVA are greatly affected by the molecular weight and hydrolysis degree of the polymer, as well as by the ratio of plasticizer incorporated (usually glycerol) and the moisture content of the film, depending on the conditioning relative humidity [42]. The film processing method (solvent casting or thermoprocessing) also affects the film’s physical properties [43]. The values of elastic modulus (EM), tensile strength (TS) and deformation at break (E%) of the obtained PVA films were in the range reported by other authors for films with similar Mw, hydrolysis degree, glycerol content and processing conditions [43]. PVA films conditioned at 53% RH were highly extensible (85%), despite their semicrystalline nature, due to the plasticized amorphous phase by the adsorbed water molecules. Incorporation of the cellulose fillers provoked the expected increase in the EM, but a decrease in the film extensibility and resistance to break. However, the relatively high values of the composite stretchability (40–50%) allow for their application as flexible packaging material. Cellulose particles bleached with sodium chlorite were slightly more effective at increasing EM than those treated with hydrogen peroxide, which could be attributed to the high crystallinity degree of these fibres and better dispersion capacity within the PVA matrix (Figure 2).

Different factors, such as and dimensions, strength, crystallinity and structure affect the reinforcing action of fibres in the composites [44]. Specifically, the reinforcing effect of cellulose fillers is greatly enhanced by increasing the aspect ratio of the particles, which improves the material’s elastic modulus and tensile strength, while reducing its extensibility. The reduction in the film extensibility is attributed to the restrictions in the polymer chain mobility imposed by the fibres and the discontinuities introduced within the polymer matrix, which increase the film fragility [52]. Other authors observed an increase in both elastic modulus and tensile strength at break while the film extensibility decreased in PVA composites with cellulose nanoparticles [50,51,53,54] or microfibrilated cellulose [55], obtained by casting. The better dispersion of cellulose nanoparticles in the water–polymer solutions, and subsequently in the composite, can explain the increase in the tensile strength. Likewise, the high aspect ratio of nanoparticles better promotes the force-transferring mechanisms in the composite [53]. However, this effect was less effective for the obtained cellulose particles due to their relatively low aspect ratio and greater size that enhances the polymer discontinuity effects in the matrix. In fact, the fibres treated with hydrogen peroxide that were more aggregated within the composited (lower aspect ratio) were less effective at reinforcing the PVA matrix than those treated with sodium chlorite.

### 2.4. Glass Transition and Crystallisation Behaviour

**Figure 5:**
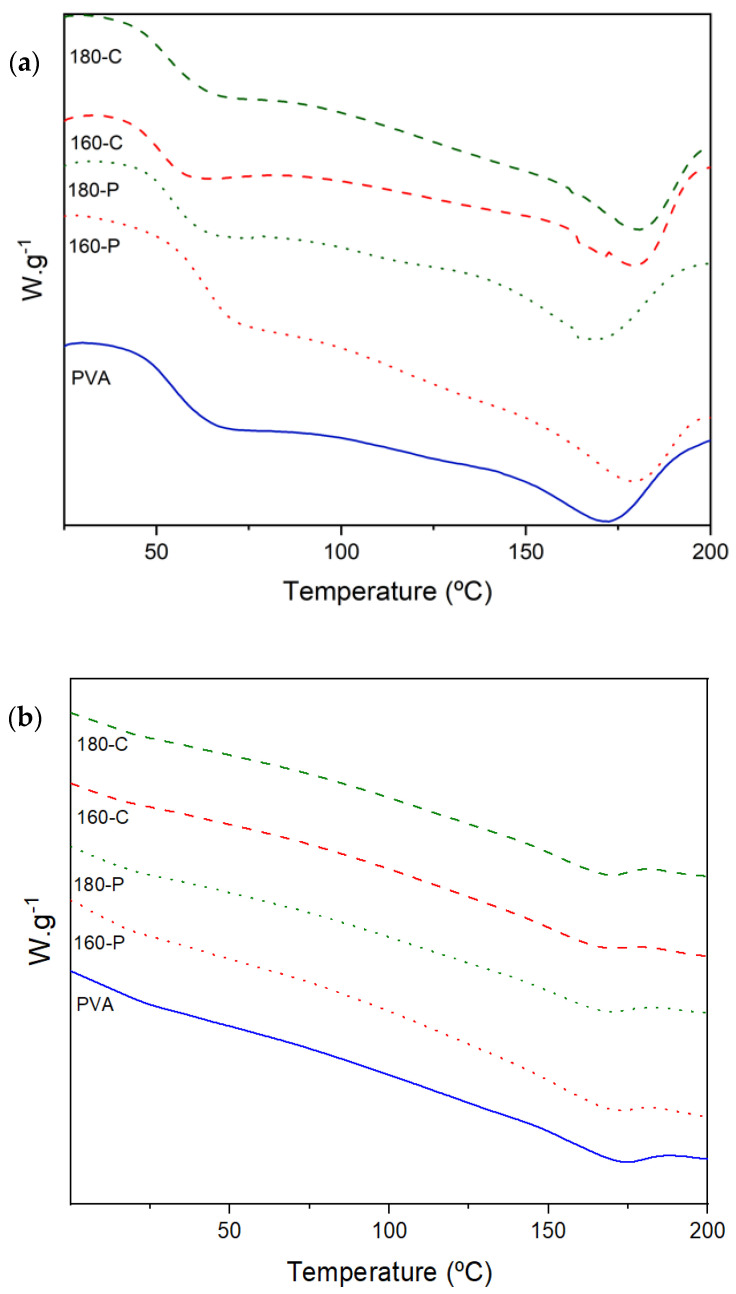
DSC thermograms (second heating step) obtained for PVA films without and with different AS cellulose particles obtained by SWE at 160 or 180 °C and bleached with hydrogen peroxide (P) or sodium chlorite (C), conditioned at 0% RH (**a**) and 53% RH (**b**).

Melting of PVA occurred in a wide range of temperatures (190 to 240 °C) as observed by other authors [56], indicating the presence of crystalline forms with very different sizes. As shown in Figure 5, the peak temperature (Tm) was about 169 (at 53% RH) and 172 °C (at 0% RH) and tended to increase when fibres were incorporated (up to 180 °C) in films conditioned at 0% RH. Crystallization of the polymer deduced from the melting enthalpy (expressed per g polymer in the sample) significantly rose as the moisture content increased (ΔHm varies from about 33 J/g in samples conditioned at 0% RH to 60 J/g in those conditioned at 53% RH), as shown in Table 5. This can be explained by the higher molecular mobility of the polymer chains at 53% RH, which favours the chain rearrangement in the more stable crystalline phase. In this sense, it is remarkable that whereas no effect of fibres was observed on PVA crystallization at 0% RH, these inhibit the PVA crystallization at 53% RH, as the ΔHm values were reduced by 23–33% in composites. This could be attributed to the blending effect, which always implies difficulties for the crystallization processes, which seems to be more marked when molecular mobility increases at high moisture content. Comparing crystallization and melting temperatures, the usual supercooling effect (ΔT) for crystallization was observed. At the lowest moisture content, fibres bleached with sodium chlorite reduced ΔT, which suggests their nucleating effect. Nevertheless, at the highest moisture content, this effect was only observed for fibre 180-C which had the highest cellulose content and crystallinity. Likewise, ΔT was lower (33 vs. 43 °C) when samples were conditioned at 53% RH, probably due to the highest molecular mobility of the polymer that implied a faster nucleating rate and crystallisation ratio. Crystalline structures act as a catalyst of the crystallisation process, reducing the crystallisation-free energy and promoting the heterogenous nucleation at higher temperatures, with a lower supercooling effect. However, the final crystallinity degree was similar in every composite.

Therefore, the increase in moisture content promoted the PVA crystallization rate while fibres inhibited the crystallization ratio in these conditions. Fibres acted as nucleating agents when they had more cellulose purity and crystallinity, favouring the crystal growth (increase in Tm) in films conditioned at 0% RH. The different crystallization degrees of the polymer will also affect both the barrier and mechanical properties of the composites.

### 2.5. Thermal Stability of the Films

**Figure 6:**
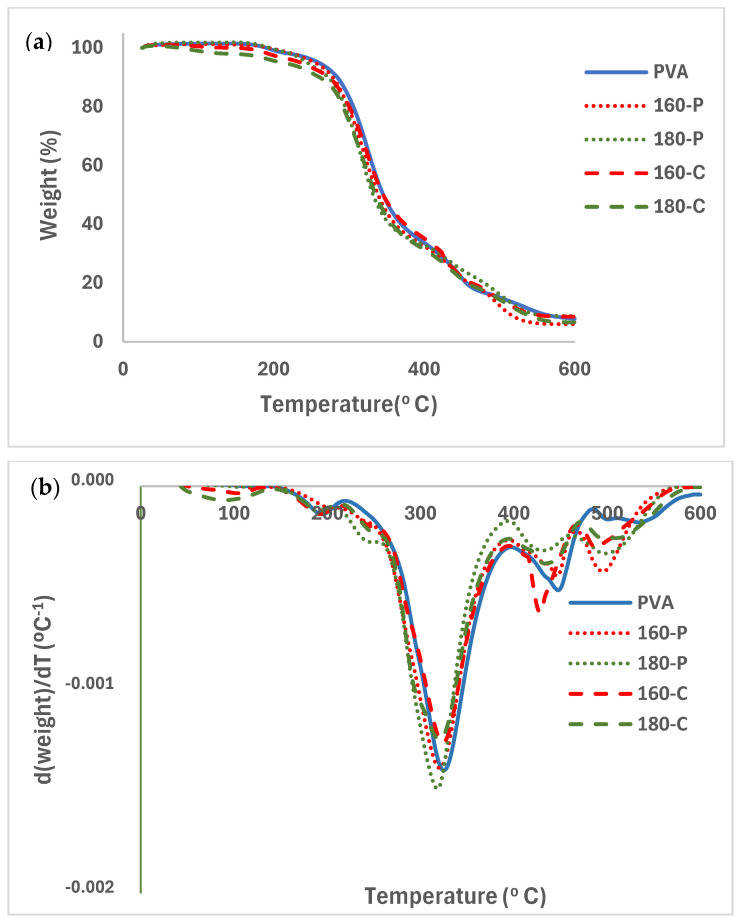
TGA (**a**) and DGTA (**b**) curves of the PVA films without and with different AScellulose particles obtained by SWE at 160 or 180 °C and bleached with hydrogen peroxide (P) or sodium chlorite (C).

The TGA and associated DTGA curves for the different PVA films with and without fibres, conditioned at 0% RH, are shown in Figure 6a and 6b, respectively). PVA films and composites exhibited a slight weight loss near 100 °C, attributed to the evaporation of bonded water. A subsequent small mass loss step occurred between 150 and 220 °C that can be attributed to the glycerol evaporation/degradation [57]. From about 220 °C, thermal degradation of PVA occurred in several steps. The first is associated with the detachment of the side chain groups (hydroxyl and acetyl) and, afterwards, the breakdown of the main polymer chains occurs, giving rise to volatile compounds such as aldehydes, ketones and carbon dioxide [47,55]. The temperature of the maximum degradation rate (Tpeak in DTGA curves) of PVA was about 324 °C. The degradation temperature of PVA was highly dependent on its molecular weight and hydrolysis degree, and the Tpeak values ranging between 250 and 315 °C, depending on its molecular characteristics [48,51]. Thermal analysis indicated that the selected PVA had a sufficient processing window (170 to 220 °C) for thermoprocessing, allowing composites to be obtained in a more industrially scalable way.

The presence of fibres hardly modified the PVA degradation pattern in the first step although small peaks in the DGTA curves were detected associated with the overlapping of the degradation peaks of the fibres components (cellulose, and residual hemicellulose and lignin) that degraded between 200 and 600 °C, with the maximum degradation rate of cellulose at 300–315 °C, and with a degradation step of the residual lignin between 350 and 500 °C [25]. Cellulose from fibres treated with hydrogen peroxide exhibited lower Tpeak values than those treated with sodium chlorite [25]. Modica et al. [17] reported two mass loss steps between 200–400 °C and 420–600 °C for pure lignin isolated from almond shell, while isolated cellulose degrades between 200 and 400 °C. 

Other studies reported an increase in the PVA degradation temperature when containing cellulose nanofibers due to the high thermal stability of the nanofibers, and the restriction of PVA chain mobility caused by a uniform distribution of the nanofibers in the matrix [58]. Nevertheless, cellulose reinforcements tend to reduce the thermal stability of other hydrophobic polymers, such as polyethylene and polypropylene, in composites [59].

## 3. Materials and Methods

### 3.1. Materials

PVA (P8136, Mw 30,000–70,000; 87–90% hydrolysed), supplied by Sigma-Aldrich, (Steinheim, Germany) was used to obtain the films, with glycerol (Sigma-Aldrich, Steinheim, Germany). Magnesium nitrate (Mg (NO_3_)_2_), phosphorus pentoxide (P_2_O_5_) from Panreac Química S.A. (Barcelona, Spain) were also used.

The cellulose fibres were obtained from almond shells (var. *Guara*) kindly supplied by Importaco SA (Valencia, Spain) from their 2022 harvest, as described by Gil-Guillén [25]. The process includes a first extraction of non-cellulosic compounds by subcritical water at 160 or 180 °C and the subsequent bleaching by two kinds of treatments: (a) hydrogen peroxide (P) at 8% (*v/v*), at 40 °C and pH 12, in four 1 h cycles with 30:1 ratio of bleaching solution, and (b) sodium chlorite (C) (1.7% *w/v*) at pH 4.5 (2 N acetate buffer) in seven 4 h cycles. Then, four kinds of fibres were used: 160-P, 160-C, 180-P and 180-C, whose composition and whiteness index are shown in Table 1, as reported by Gil-Guillén et al. [25].

### 3.2. Obtaining Composites

PVA with molecular characteristics (Mw and degree of hydrolysis) that allowed high mechanical and barrier performance and sufficient thermoprocessing window (difference between melting and degradation temperatures) was chosen. The PVA and fibres (10% wt. in the mixture) were pre-mixed in powder after conditioning at 0% relative humidity (RH) in a desiccator containing P_2_O_5_ for 2 weeks. The composite blends were prepared by melt blending, in an internal mixer (HAAKETM PolyLabTM QC, Thermo Fisher Scientific, Karlsruhe, Germany), using glycerol as a plasticiser at 10% in the blend. Melt blending was carried out at 180 °C and 50 rpm for 6 min, while the torque was measured throughout the mixing time and this reached a constant value, indicating a homogenous blending. The obtained blends were cooled at room temperature for 1 h and cold powdered in a Thermomix TM-5 (Vorwerk Spain M.S.L., S.C., Madrid, Spain) with liquid N_2_. Then, films (4 g per film) were thermoformed using a hot-plate hydraulic press (model LP20, Labtech Engineering, Thailand), applying 3 min preheating at 180 °C, 3 min compression at 100 bar and 180 °C, and cooling to 80 °C for 3 min. The obtained films were conditioned at 0 or 53% RH in desiccators, containing P_2_O_5_ or Mg (NO_3_)_2_ oversaturated solution, respectively, previously to the corresponding analysis.

### 3.3. Composite Properties

#### 3.3.1. Microstructure

The microstructure of the cross-sections of the films, conditioned at 0% RH, was examined by high-resolution FESEM. The films were cryofractured in slush nitrogen, coated with platinum using an EM MED020 sputter coater (Leica BioSystems, Barcelona, Spain), and then observed using a field emission scanning electron microscope equipped with a focused ion gun (FESEM Ultra 55, Zeiss, Oxford Instruments, Oxford, UK) at an accelerating voltage of 2.0 kV.

#### 3.3.2. Optical Properties: Colour and Transparency

The reflection spectra of films conditioned at 53% RH were obtained, between 400 and 700 nm, using a spectropolarimeter (CM-3600d Minolta CO., Tokyo, Japan), with a white background with known reflectance (Rg) and a black background (R_0_). Using the Kubelka–Munk scattering theory, Equations (1)–(3) were used to determine the reflectance of a film of infinite thickness (R_∞_). The CIE Lab* colour coordinates were determined from the R_∞_ spectra, using a 10° observer and D65 illuminant. The psychometric coordinates hue (h_ab_*) (Equation (4)) and chroma (C_ab_*) (Equation (5)) were calculated from the a* and b* values. Additionally, the colour difference (ΔE *) between the composite films and the PVA control films (Equation (6)) was determined.

(1) $$R_{\infty }=a-b$$

(2) $$a=\frac{1}{2}\left[R+\left(\frac{R_{0}-R+R_{g}}{R_{0}\times R_{g}}\right)\right]$$

(3) $$b=\sqrt{a^{2}-1}$$

(4) $$h_{ab}^{*}=arctg\left(\frac{b^{*}}{a^{*}}\right)$$

(5) $$C_{ab}^{*}=\sqrt{a^{*2}+b^{*2}}$$

(6) $${∆E}^{*}=\sqrt{{\left({∆L}^{*}\right)}^{2}+{\left({∆a}^{*}\right)}^{2}+{\left({∆b}^{*}\right)}^{2}}$$

The UV-Visible transmittance spectra of the different films were also obtained by using the spectrophotometer, model UV-3600 i Plus (Shimadzu, Tokyo, Japan) from 200 to 800 nm. For each spectrum, a representative average of 3 scans was obtained using a data pitch of 0.5 nm, a bandwidth of 2.0 nm, and a scanning speed of 100 nm/min.

#### 3.3.3. Thermal Behaviour (DSC and TGA)

The thermal stability of the films was measured with a thermogravimetric analyser (TGA 1 Stare System, Mettler-Toledo Inc., Greifensee, Switzerland). Samples (3–5 mg) were placed in an alumina crucible and heated from 25 °C to 600 °C at a rate of 10 °C/min with a nitrogen flow of 10 mL/min. The STARe evaluation software (version V12.00a, Mettler-Toledo, Switzerland) provided DTGA curves, allowing the determination of the initial and peak degradation temperature. Analyses were performed in duplicate for each film sample conditioned at 0% RH.

DSC analysis was performed with a differential scanning calorimeter (Stare System, Mettler-Toledo Inc., Switzerland). Film samples weighing 3–5 mg were placed in aluminium pans and subjected to a five-step programme under nitrogen flow (30 mL/min). Heating started from 20 °C to 210 °C at 10 °C/min, with a 1 min hold at 210 °C, followed by cooling to 20 °C at 10 °C/min, with 1 min hold, and reheating to 210 °C at 10 °C/min. Duplicate tests were performed for each film formulation conditioned at 0% RH as well as for those conditioned at 53% RH. From the second heating step, the glass transition temperature (Tg), and melting temperature (Tm) and enthalpy (ΔHm) were obtained, as well as the crystallisation temperature (Tc) from the cooling step.

#### 3.3.4. Mechanical Properties

Tensile properties of the films conditioned at 53% RH, such as elastic modulus (EM), tensile strength (TS) and elongation at the breaking point (E), were analysed from the stress (σ) vs. Hencky strain (ε_H_) curves, following the ASTM D882-02 standard [60]. Film strips of 25 mm × 100 mm were mounted in the grips (50 mm separation) of the universal testing machine (Stable Micro Systems, TA. XT plus, Haslemere, UK) and traction was applied at 50 mm/min until the specimens broke. Measurements were performed on six samples per film formulation.

#### 3.3.5. Barrier Properties

Water vapour (WVP) and oxygen (OP) permeability were measured in the different films conditioned at 53% RH. WVP was measured using a gravimetric method based on ASTM E96/E96M [61] considering the correction of McHugh et al. [62]. The 3.5 cm-diameter films were sealed in Payne permeability cups (Elcometer SPRL, Hermelle/s Argenteau, Belgium) containing 5 mL of distilled water and placed in desiccators at 25 °C and 53% RH, thus creating a RH gradient of 100–53% through the films. For 27 h, a precision balance (ME36S, Sartorius, ±0.0001 g) was used to record the weight of the cups every 75 min. The slope of the weight loss vs. time curve, once the steady state was reached allows for the estimation of the water vapour transmission rate that was used to calculate the WVP, considering the film thickness and the relative humidity gradient. Three duplicates of the analysis were made per film formulation

According to ASTM D3985-05 [63], the oxygen permeability of the films (50 cm^2^ area) was assessed, using Systech Illinois, Model 8101e equipment (Illinois, IL, USA). The oxygen transmission rate (OTR) was obtained when the steady state was reached. The OP was calculated from the OTR, the film thickness and the oxygen partial pressure gradient through the film. Three replicates per film formulation were carried out. The films’ thickness was determined by using a digital micrometre (Palmer, COMECTA, Barcelona, Spain) in at least six random positions.

#### 3.3.6. Moisture Content

The equilibrium moisture content of the films conditioned at 53% RH was assessed by gravimetric analysis. The conditioned samples were weighed (Wm) and dried in a laboratory oven at 105 °C for 24 h till constant weight and conditioned at 0% RH before weighing (Wd) (NSW-143, Narang Scientific Works Pvt. Ltd., New Delhi, India). The equilibrium percentage of moisture content (dry basis) was determined by (Equation (7)).

(7) $$\mathrm{Moisture}\;\mathrm{content}\;(\%)=\frac{\left(Wm-Wd\right)}{Wd}\times 100$$

#### 3.3.7. Antioxidant Activity

The potential antioxidant capacity of the films was measured by applying the adapted method of ABTS (2,2′-azinobis-(3-ethylbenzothiazoline-6-sulfonic acid)), described by Nunes et al. [64]. The formation of ABTS∙+ was produced by reacting ABTS (7 mM) with potassium persulphate (2.45 mM) in the dark, at room temperature, for 16 h. The ABTS∙+ solution (1 mL) was diluted in ethanol (about 80 mL) to achieve an absorbance between 0.700 and 0.800 at 734 nm, measured with a spectrophotometer (Jenway 6405 UV/Vis, Stone, UK). Each film sample (1 cm^2^) was immersed in 1.5 mL of the ABTS∙+ solution and orbital stirred at 80 rpm in the dark and the absorbance at 734 nm was measured after different reaction times (Af). The ABTS∙+ solution without film was used as blank (Ab) at different reaction times. The assay was performed in triplicate for each film. The antioxidant activity of the film was determined through the inhibition percentage determined by (Equation (8)) at the different reaction times.

(8) $$\mathrm{Inhibition}\;(\%)=100\;\times \;\frac{Ab-Af}{Ab}$$

#### 3.3.8. Contact Angle

The static water contact angles (WCAs) of the films were measured by the Sessile drop method with ultrapure water (Milli-Q), using a contact angle instrument (OCA 20, Dataphysics, Valbom, Portugal) with automatic image capture analysis (Dataphysics SCA20_ M4), at room temperature. The water drop (3 µL) was applied onto the film surface and the image was captured and analysed for 10 s. At least four droplet images were taken for each film sample.

#### 3.3.9. Statistical Analysis

The statistical analysis of the data was performed through analysis of variance (ANOVA) using Statgraphics Centurion XVII-X64 and Fisher’s Least Significant Difference at the 95% confidence level.

## 4. Conclusions

PVA composite films containing cellulose fibres, obtained from almond shell by applying subcritical water extraction and different bleaching treatments, could be successfully prepared by melt blending and compression moulding. The selected polymer (Mw 30,000–70,000; 87–90% hydrolysed) exhibited good thermal stability above the softening temperature, offering a processing window of 170–220 °C. Dispersion of cellulose fibres in composites was better in those treated with sodium chlorite than in those bleached with hydrogen peroxide due to the different morphological and compositional characteristics. Residual phenolic compounds in the fibres provide the composite with antiradical activity; the higher the residual lignin, the greater the ABTS∙+ inhibition ratio. Both the presence of phenolic compounds and dispersed cellulose particles reduced the film transparency, mainly in the UV range making these more appropriate for packaging of food sensitive to oxidation. Fibres reduced the oxygen permeability of the PVA films, mainly (by 75%) those SWE treated at 160 °C that contained more hemicellulose. Permeability to water vapour was also reduced by fibres bleached with oxygen peroxide. All fibres increased the elastic modulus of the composites, especially those treated with sodium chlorite with the highest crystallinity values. Fibres also interfered with the PVA crystallisation, depending on the film moisture content and molecular mobility. The fibres with the highest cellulose purity and crystallinity acted as nucleating agents, favouring the crystal growth (increase in Tm) in films conditioned at 0% RH, where PVA crystallization progressed to a lower extent. Therefore, cellulose fibres obtained from almond shells by applying greener methods, such as subcritical water extraction combined with a bleaching step, could be used to obtain PVA biocomposites, lowering the material cost, and providing them with antioxidant capacity and improved elastic modulus and barrier properties. Differences in cellulose purification conditions allowed the final properties of composites to be modulated, whereby their selection would depend on the requirements of the films for their final application in food packaging.

## Figures and Tables

**Table 1:** Content (g/100 g sample) of cellulose, hemicellulose, lignin and ashes and whiteness index (WI) of fibres obtained in the combined treatments of SWE at 160 or 180 °C and bleaching with hydrogen peroxide (160-P and 180-P) or sodium chlorite (160-C and 180-C). Values of the raw AS are also included. Data from Gil-Guillen et al. [25].

Fibre	Cellulose	Hemicellulose	Lignin	Ashes	WI
AS	26.8 ± 1.3	23.6 ± 0.2	21.2 ± 2.0	2.0 ± 0.2	-
160-P	70.5 ± 0.9 ^b^	20.3 ± 1.0 ^a^	8.5 ± 2.0 ^a^	4.3 ± 0.3 ^a^	61.7 ± 0.0 ^d^
180-P	78.4 ± 0.2 ^a^	12.2 ± 1.2 ^b^	4.9 ± 1.2 ^ab^	4.1 ± 0.3 ^a^	68.5 ± 0.1 ^c^
160-C	77.0 ± 4.0 ^a^	20.0 ± 2.0 ^a^	3.5 ± 0.5 ^b^	1.7 ± 0.3 ^c^	72.4 ± 0.0 ^b^
180-C	83.7 ± 2.4 ^a^	12.5 ± 0.7 ^b^	2.7 ± 0.6 ^b^	1.1 ± 0.1 ^c^	79.3 ± 0.4 ^a^

Different letters in superscript indicate significant differences between fibre samples (*p* < 0.05).

**Table 2:** Colour coordinates, lightness, chrome and hue (L, C_ab_*, h_ab_*) of the PVA films without and with different AS cellulose particles obtained by SWE at 160 or 180 °C and bleached with hydrogen peroxide (P) or sodium chlorite (C). The total colour difference (ΔE) of composites with respect to PVA films is also included.

Sample	L	C_ab_*	h_ab_*	ΔE*
PVA 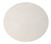	73.2 ± 0.9 ^a^	16.0 ± 3.0 ^d^	46.0 ± 0.1 ^b^	^-^
160-P 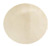	55.2 ± 0.3 ^d^	52.9 ± 0.0 ^a^	73.3 ± 0.0 ^a^	24.0 ± 0.9 ^a^
180-P 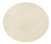	53.7 ± 0.9 ^d^	51.0 ± 1.0 ^a^	73.4 ± 0.3 ^a^	24.9 ± 2.2 ^a^
160-C 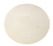	62.9 ± 0.5 ^b^	39.1 ± 0.8 ^c^	74.0 ± 0.4 ^a^	17.5 ± 0.7 ^b^
180-C 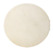	60.1 ± 0.1 ^c^	43.9 ± 1.2 ^b^	73.6 ± 0.3 ^a^	20.0 ± 0.7 ^b^

Different letters in superscript indicate significant differences between samples (*p* < 0.05).

**Table 3:** Equilibrium moisture content, contact angle and water vapour (WVP) and oxygen (OP) permeability of the PVA films without and with different AS cellulose particles obtained by SWE at 160 or 180 °C and bleached with hydrogen peroxide (P) or sodium chlorite (C).

Sample	Moisture (g/100 g Solids)	Contact Angle (^o^)	OP × 10^14^ (cm^3^/m s Pa)	WVP × 10^11^ (g/Pa s m)
PVA	12.1 ± 1.1 ^a^	44 ± 12 ^a^	11.5 ± 0.8 ^a^	219 ± 5 ^b^
160-P	7.0 ± 0.8 ^b^	52 ± 13 ^a^	3.0 ± 0.6 ^c^	162 ± 3 ^d^
180-P	10.8± 1.6 ^ab^	48 ± 16 ^a^	4.3 ± 0.2 ^c^	182 ± 6 ^c^
160-C	10.0 ± 4.0 ^ab^	56 ± 11 ^a^	2.8 ± 0.1 ^c^	267 ± 14 ^a^
180-C	10.0 ± 3.0 ^ab^	42 ± 10 ^a^	6.9 ± 1.0 ^b^	211 ± 12 ^b^

Different letters in superscript indicate significant differences between samples (*p* < 0.05).

**Table 4:** Tensile parameters (elastic modulus of elasticity: EM, and stress: TS and strain: E% at the break) of the PVA films without and with different AS cellulose particles obtained by SWE at 160 or 180 °C and bleached with hydrogen peroxide (P) or sodium chlorite (C).

Sample	EM (MPa)	TS (MPa)	E (%)
PVA	123 ± 7 ^c^	44.0 ± 7.0 ^a^	85.1 ± 0.6 ^a^
160-P	150 ± 20 ^ab^	17.1 ± 1.6 ^b^	51.3 ± 0.3 ^b^
180-P	160 ± 8 ^ab^	18.7 ± 0.9 ^b^	44.0 ± 14 ^b^
160-C	185 ± 9 ^a^	18.7 ± 2.9 ^b^	40.0 ± 12 ^b^
180-C	191 ± 18 ^a^	19.4 ± 2.5 ^b^	49.0 ± 5.0 ^b^

Different letters in superscript indicate significant differences between samples (*p* < 0.05).

**Table 5:** Glass transition temperature, crystallization and melting temperatures and melting enthalpy of PVA in films without and with different AS cellulose particles obtained by SWE at 160 or 180 °C and bleached with hydrogen peroxide (P) or sodium chlorite (C).

Sample	Tg (°C)	Tc (°C)	Tm (°C)	ΔHm (J/g)
	0% RH			
PVA	54.5 ± 0.4 ^a^	129.2 ± 0.5 ^c^	172.0 ± 1.0 ^b^	33.0 ± 3.0 ^a^
160-P	55.0 ± 4.0 ^a^	139.0 ± 5.0 ^ab^	178.0 ± 1.0 ^a^	34.0 ± 4.0 ^a^
180-P	57.0 ± 2.0 ^a^	131.6 ± 0.5 ^bc^	174.0 ± 2.0 ^b^	31.2 ± 1.2 ^a^
160-C	54.0 ± 3.0 ^a^	146.0 ± 2.0 ^a^	180.0 ± 1.3 ^a^	37.7 ± 1.2 ^a^
180-C	54.3 ± 1.7 ^a^	145.0 ± 2.0 ^a^	180.0 ± 1.2 ^a^	33.0 ± 3.0 ^a^
	53% RH			
PVA	11.8 ± 1.7 ^a^	136.0 ± 4.0 ^a^	169.0 ± 2.0 ^a^	60.0 ± 6.0 ^a^
160-P	10.7 ± 0.7 ^a^	126.1 ± 0.8 ^a^	164.0 ± 5.0 ^a^	46.0 ± 3.0 ^b^
180-P	12.2 ± 1.8 ^a^	131.0 ± 5.0 ^a^	164.5 ± 1.7 ^a^	42.0 ± 4.0 ^b^
160-C	14.5 ± 0.2 ^a^	131.0 ± 3.0 ^a^	165.1 ± 1.4 ^a^	39.4 ± 0.7 ^b^
180-C	13.5 ±3.3 ^a^	137.0 ± 1.0 ^a^	166.3± 0.2 ^a^	40.4 ± 1.0 ^b^

Different letters in superscript indicate significant differences between samples (*p* < 0.05).

## Data Availability

The original contributions presented in the study are included in the article, further inquiries can be directed to the corresponding author.
